# Insights into metabolic changes during epidermal differentiation as revealed by multiphoton microscopy with fluorescence lifetime imaging

**DOI:** 10.1038/s41598-025-90101-4

**Published:** 2025-02-21

**Authors:** Monika Malak, Chen Qian, Jeemol James, Syam Nair, Julie Grantham, Marica B. Ericson

**Affiliations:** 1 Department of Chemistry and Molecular Biology, Faculty of Science, University of Gothenburg, Gothenburg, 412 96 Sweden; 2 Institute of Neuroscience and Physiology, The Sahlgrenska Academy, University of Gothenburg, Gothenburg, 413 90 Sweden; 3 Institute of Clinical Sciences, The Sahlgrenska Academy, University of Gothenburg, Gothenburg, 416 85 Sweden

**Keywords:** Multiphoton microscopy, Fluorescence lifetime imaging, Epidermal differentiation, Metabolism, Label-free imaging, Cellular imaging, Fluorescence imaging

## Abstract

Rapid developments in the field of organotypic cultures have generated a growing need for effective and non-invasive methods for quality control during tissue development. In this study, we correlate metabolic changes with epidermal differentiation and demonstrate that multiphoton microscopy with fluorescence lifetime imaging (MPM-FLIM) can be applied to monitor epidermal differentiation of keratinocytes with respect to proliferative and differentiated states. In a 2D keratinocyte tissue culture model, increased expression of differentiation markers keratin-1 and keratin-10 was induced with calcium supplementation. An accompanying shift from glycolysis to mitochondrial respiration was detected in metabolic flux assays. Analysis of MPM-FLIM images acquired at 750 nm and 900 nm excitation revealed a decreased relative fraction of intracellular NADH and FAD after high calcium treatment, consistent with increased oxidative phosphorylation. Epidermal differentiation could be monitored over a 96 h period. Discrimination analysis based on k-means clustering generated clusters that correlated well with the duration of high Ca^2+^ treatment, suggesting that MPM-FLIM can provide useful parameters for monitoring keratinocyte differentiation.

**Supplementary Information:**

The online version contains supplementary material available at 10.1038/s41598-025-90101-4.

## Introduction

The development of organotypic cell cultures has revolutionized biomedical research by providing an in vitro model system that closely mimics the complex cellular organization and microenvironment of native tissues^1–5^. These cultures offer invaluable platforms for studying cellular behavior, physiological processes, and disease mechanisms, as well as for drug discovery and regenerative medicine applications. Skin is one of the tissue types that has attracted much attention in the field, especially in the development of more realistic skin models^6–8^, including the recent publication of a 3D bioprinted skin model incorporating hair follicles^9^. However, the success of organotypic cultures depends on their fidelity in recapitulating the native tissue characteristics, necessitating stringent quality control measures at all stages of culture establishment and maintenance. Traditional biochemical and immunohistological approaches are often time consuming and destroy the sample. Consequently, there is an increasing need for efficient and reliable methods for assessing the developmental state of cell cultures in a non-invasive manner with minimal disturbance to the samples.

Multiphoton laser scanning microscopy (MPM) has emerged as a powerful tool for non-invasive and label-free imaging of biological samples with high spatial resolution, deep tissue penetration and reduced photobleaching and phototoxicity compared to confocal microscopy. The technique utilizes femtosecond pulsed lasers to excite fluorophores in a small focal volume through the simultaneous absorption of two or more photons^10^. It can be combined with an additional technique known as time-correlated single photon counting (TCSPC), which records, with picosecond precision, the arrival time of each photon at the detector relative to the excitation pulse^11^. One can then reconstruct the fluorescence decay in a particular sample or image region by pooling together the arrival times of the corresponding photons over many excitation pulses. From this, we can extract the excited state lifetime of the fluorophore, typically by fitting to an exponential decay model or through a method known as phasor analysis^12,13^. When used in the context of microscopy, this technique is known as fluorescence lifetime imaging microscopy (FLIM), and we refer to the variant using multiphoton excitation as MPM-FLIM.

MPM-FLIM has been proposed as a tool for assessing the cellular metabolic state by imaging the autofluorescence of key metabolic coenzymes nicotinamide adenine dinucleotide (NADH) and flavin adenine dinucleotide (FAD), in, e.g., cardiomyocytes^14^, and epithelial cells^15^. During glycolysis and the Krebs cycle, the non-fluorescent NAD^+^ is reduced to the fluorescent NADH, and the fluorescent FAD is reduced to the non-fluorescent FADH_2_. These reduced coenzymes are oxidized in the mitochondrial complexes to produce ATP via oxidative phosphorylation. Changes in the relative rates of glycolysis and oxidative phosphorylation affects the ratio of the oxidized and reduced forms of the coenzymes. Glycolytic cells would have high production and circulation of NADH in the cytoplasm, whereas cells relying more on oxidative phosphorylation would have a decreased NADH/FAD ratio due to increases in the rates of oxidation of NADH to NAD + and FADH_2_ to FAD. As a result, the NADH/FAD ratio has long been suggested as a marker of cellular metabolism, with most existing methods focusing on the calculation of a single numerical parameter to quantify the cellular redox state based on fluorescence intensity^16–18^ or fluorescence lifetime^19,20^. However, such parameters are not always indicative of the state of cellular metabolism^21,22^. Additionally, distinct fluorescence lifetimes have been attributed to the free and protein-bound forms of NADH and FAD, and therefore the fractional components of the intracellular autofluorescence decays could also be affected by changes in the cellular metabolic state^23^. As such, interpretation of fluorescence decay in the context of cellular autofluorescence can be complex. In our previous work, we demonstrate that spectrally overlapping emissions from structural proteins such as keratin should be taken into account in the analysis of autofluorescence in skin tissue^24^. Careful choice of experimental parameters and analysis methodology are therefore critical for the MPM-FLIM approach in the context of monitoring cellular metabolism.

When mimicking human epidermis in vitro the understanding of cell differentiation is important. Keratinocytes, being the dominant epidermal cell type, continuously proliferate in *stratum basale* (SB) and commit to vertical migration towards the surface of the skin to form a stratified epidermal tissue layer^25^. Eventually, cells flatten out forming the cornified skin layer *stratum corneum* (SC), which consists of tightly packed dead keratinocytes with a lipid matrix in-between forming a protective barrier from the external environment^26^. The cornified skin layer is continuously replenished with the next generation of cells migrating from the SB. Differentiation has generally been associated with a shift from glycolysis to mitochondrial oxidative metabolism^27^, but the evidence mostly comes from less differentiated cell types, such as in induced pluripotent stem cells differentiating into fibroblast and keratinocyte progenitors^28^ as well as cardiomyocytes^29^, or when reprogramming skin fibroblasts into induced pluripotent stem cells^30^. It is therefore unclear whether this relationship holds true for epidermal tissue, with existing studies providing only mixed, indirect indications^31,32^. While the production of reactive oxygen species from mitochondrial respiration is required for keratinocyte differentiation^31^, the activation of autophagy genes during keratinocyte differentiation has been associated with a decrease in the number of mitochondria^32^. To our knowledge, there has been no direct observation of the rates of glycolysis and oxidative phosphorylation during keratinocyte differentiation into terminally differentiated cells. Therefore, in order to implement MPM-FLIM as a method to monitor epidermal tissue cultures, the link between metabolic status and epidermal differentiation needs to be first confirmed.

In this study, we characterize a keratinocyte tissue culture model with respect to its differentiation and metabolic states to establish a sound basis for the interpretation of MPM-FLIM data. A previously established 2D keratinocyte culture model was chosen for its robustness^33,34^. Cell differentiation was investigated by qPCR, immunoblotting and immunofluorescence for tissue cultures at different Ca^2+^ levels, and confirmed to take place at 1.5 mM Ca^2+^. A metabolic flux assay revealed that cultures undergoing differentiation exhibited a shift in metabolism from glycolysis to oxidative phosphorylation. We further demonstrate that this metabolic change can be detected using MPM-FLIM and proceed to follow the differentiation process in keratinocytes over a 96 h period. K-means clustering on MPM-FLIM data acquired from tissue cultures generated clusters that correlated with the duration of high Ca^2+^ treatment. Our results suggest that keratinocytes undergoing differentiation exhibit a shift in cellular metabolism that can be monitored using MPM-FLIM.

## Results

### Confirming keratinocyte differentiation using high calcium supplementation

To confirm that high Ca^2+^ treatment leads to cell differentiation in our keratinocyte model, we performed immunofluorescence assays to identify key differentiation markers, and further validated the results with western blotting and qPCR. Keratins are important markers of epidermal differentiation^35^. The dimers of type I and type II keratins form tetrameric structures that then assemble into a complex network of intermediate filaments, providing the cell with mechanical strength and resilience^36^. Differentiation of keratinocytes is associated with a change in the dominant keratin isoforms that make up the intermediate filaments. The proliferating basal layer of the epidermis is dominated by type II keratin 5 (K5) and type I keratin 14 (K14), and the suprabasal layers of differentiating cells are abundant in type II keratin 1 (K1) and type I keratin 10 (K10)^37^. Furthermore, extracellular calcium has been reported to affect desmosome formation^38^, which is directly connected to the expression of K1 and K10^39^.

**Fig. 1:**
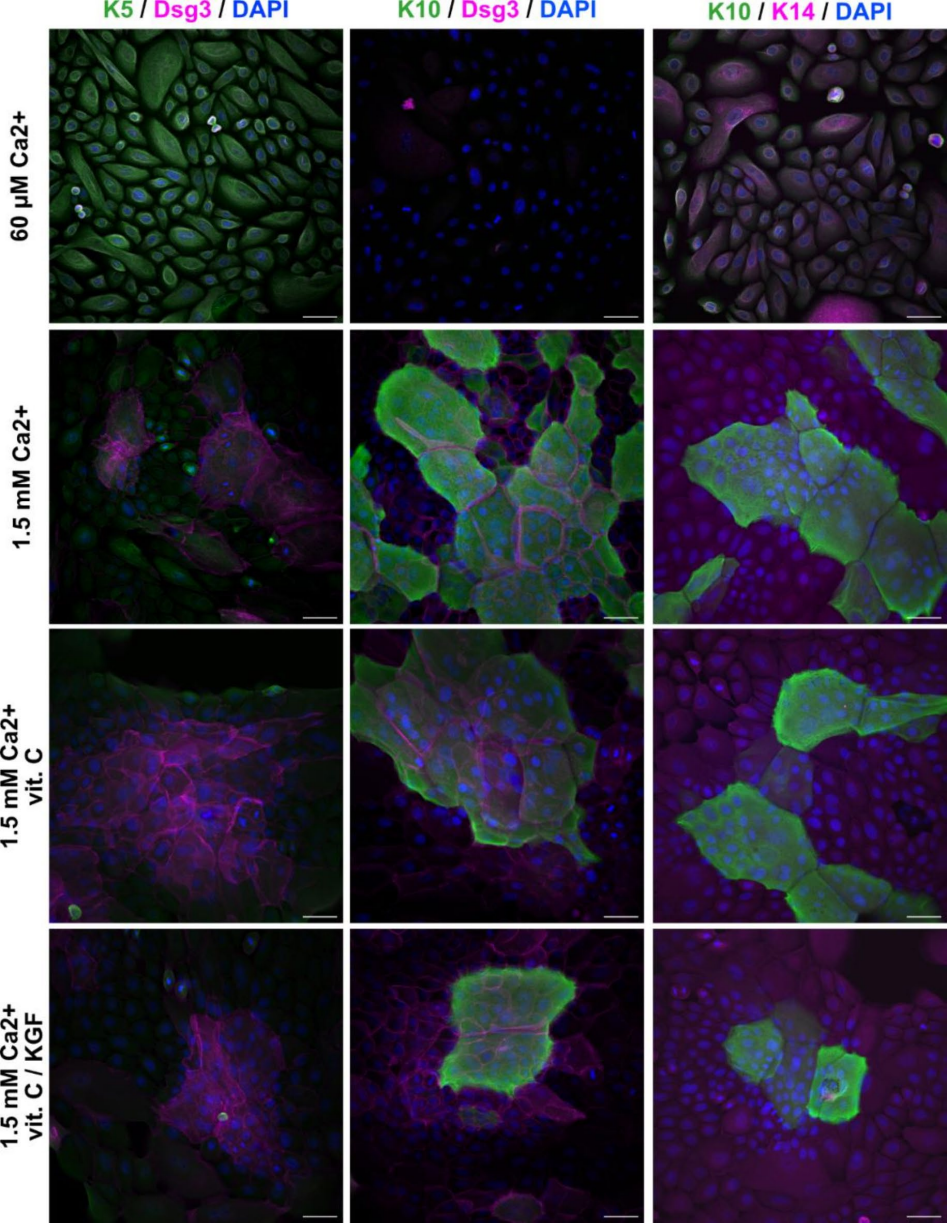
High calcium treatments induce the expression of K10 and desmosome formation, initial markers of epidermal differentiation. Confocal microscopy images of keratinocytes treated with (top to bottom): regular growth medium, 1.5 mM Ca^2+^, 1.5 mM Ca^2+^ and vitamin C, and 1.5 mM Ca^2+^, vitamin C and KGF. After 96 h of treatment, cells were stained for (left to right): K5/Dsg3/DAPI, /K10/Dsg3/DAPI, and K10/K14/DAPI. Primary antibodies were incubated simultaneously, as were the secondary antibodies. The images shown are the maximum projections of four different focal planes (z = 4 μm, z = 7 μm, z = 10 μm, and z = 13 μm). Scale bar: 50 μm. Images were created using ImageJ (v1.54, https://imagej.net/software/fiji/).

Neonatal human epidermal keratinocytes (HEKn) were cultured at two different Ca^2+^ levels, i.e., low (60 µM) and high (1.5 mM) Ca^2+^, to maintain proliferative potential or to stimulate differentiation respectively. In addition to varying Ca^2+^ levels, the effect of vitamin C and keratinocyte growth factor (KGF), recommended supplementation for 3D cultures of reconstructed human epidermis^7^, was evaluated for the high Ca^2+^ cultures. After 96 h of treatment, the samples were fixed and immunostained for the keratin isoforms K5, K10 and K14 as well as desmoglein-3 (Dsg3) to confirm desmosome formation. The samples were additionally stained with DAPI to identify cell nuclei. Confocal fluorescence microscopy images acquired from a set of cell cultures are presented in Fig. 1. The immunofluorescence assay confirmed that high Ca^2+^ treatment, alone and with additional supplementation of vitamin C and KGF, led to desmosome formation (Dsg3 positive) and the presence of K10-positive cells, whereas K5 and K14 (markers for proliferation) were dominant in control cells at low Ca^2+^ (60 µM). K14-positive cells (magenta) were additionally observed surrounding the differentiated cells stained for K10 (green) in high calcium treatments. A more detailed analysis of the confocal microscopy data at 4 different focal planes within the sample (4 μm, 7 μm, 10 μm, 13 μm) showed that cells positive for K10 were located suprabasally, while Dsg3 stained cells were evenly distributed within the sample (Fig. S2). This indicates that high Ca^2+^ treated samples contain a heterogenous cell population that consists of both proliferative and differentiated cells in different layers and is an important consideration when moving towards MPM-FLIM analysis targeting proliferative vs. differentiated cells.

We also performed western blotting and qPCR on the lysates of similarly cultured cells (Fig. S1). The production of K1 and K10 in high Ca^2+^ cultures was confirmed (Fig. S1a). The qPCR analysis showed that the expression of K1 and K10 genes increased over 20-fold in cells grown in high Ca^2+^ medium, in comparison to regular growth medium (low Ca^2+^) (Fig. S1b, c). Surprisingly, additional supplementation with Vitamin C and KGF did not further increase K1 and K10 expression, despite their proposed involvement in promoting cell proliferation and migration^7^. In fact, the supplementation of Vitamin C and KGF in combination resulted in slightly lower levels of K1 and K10 expression compared to cells grown in high Ca^2+^ medium without additional supplementation. We additionally assessed changes in the expression of K6, a marker of hyperproliferative cells^37^, found to be similar across all treatment conditions. Our interpretation is that cells grown in a 2D cell culture exhibit a hyperproliferative state in general, albeit the cells are differentiating.

### Differentiation is associated with a metabolic shift towards oxidative phosphorylation

**Fig. 2:**
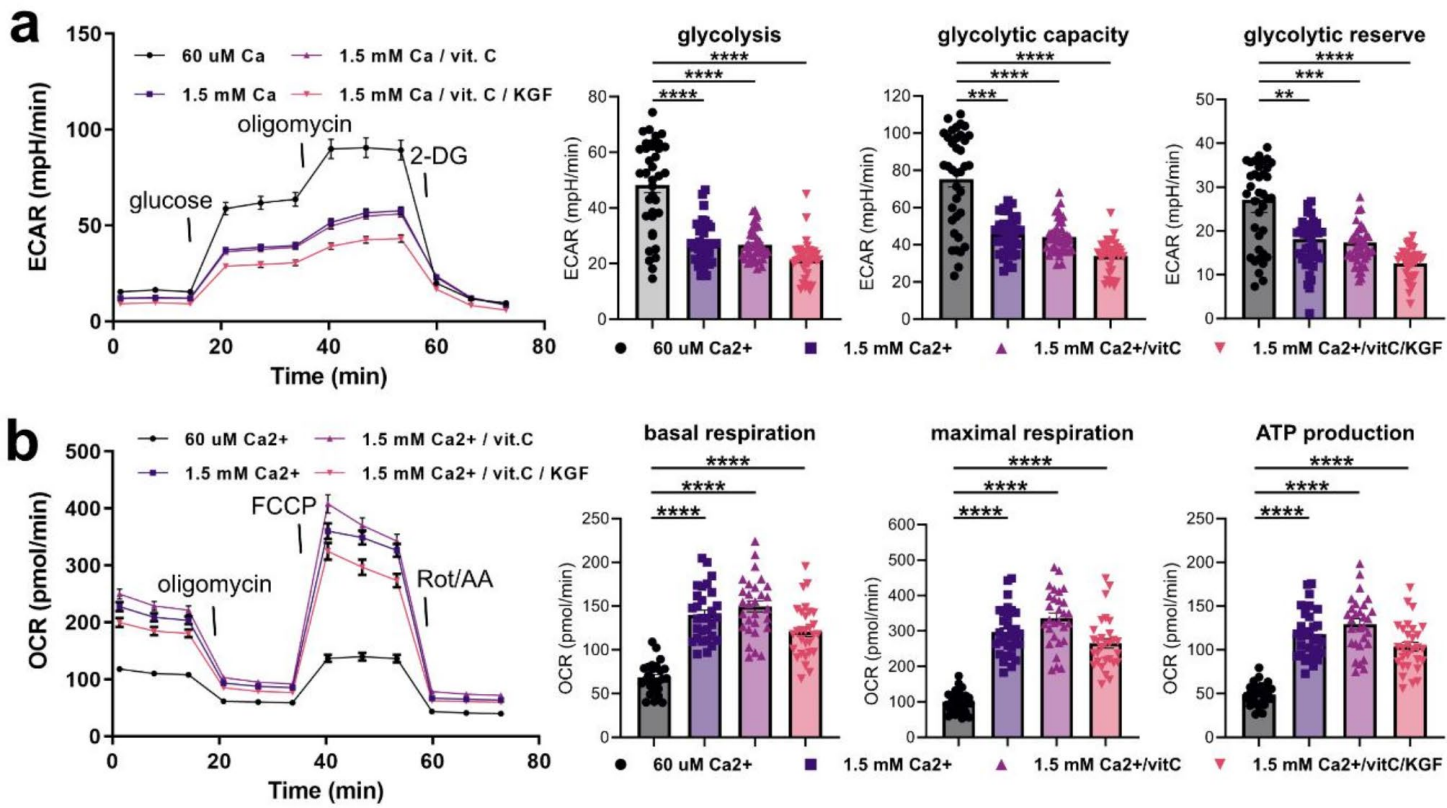
High calcium treatments induce a shift from glycolysis to mitochondrial respiration. Measurement and quantification of (**a**) ECAR and (**b**) OCR assessed using a Seahorse XFe96 Extracellular Flux Analyzer on keratinocytes treated for 96 h with regular growth medium, 1.5 mM Ca^2+^, 1.5 mM Ca^2+^ and vitamin C, 1.5 mM Ca^2+^, vitamin C and KGF. *p ≤ 0.05, **p ≤ 0.01; ****p ≤ 0.0001. Statistics compared by Kruskal–Wallis followed by Dunn’s multiple comparisons. The data was normalized to total protein levels as measured by the spectrophotometric readout from the Pierce BCA assay. Markers on bar plots correspond to individual experimental replicates.

Having confirmed that high Ca^2+^ treatment produced the expected differentiation markers in the keratinocyte culture, we proceeded to investigate how metabolic changes are associated with the differentiation process. The metabolism of keratinocytes was investigated with a Seahorse XFe96 Flux Analyzer (Fig. 2). A Glycolysis Stress Test confirmed that the proliferative cells grown in 60 µM Ca^2+^ medium exhibited higher glycolytic activity than those grown at 1.5 mM Ca^2+^, seen as a higher extracellular acidification rate (ECAR), related to the proton production during glycolysis (Fig. 2a). The quantification of ECAR showed a significantly higher glycolysis, glycolytic capacity and glycolytic reserve in the keratinocytes grown in low calcium in comparison to the high calcium treatments. In agreement with the ECAR readout, a Mito Stress Test demonstrated a significantly higher oxygen consumption rate (OCR) in the high calcium-treated cells, indicating a higher rate of oxidative phosphorylation (Fig. 2b). The quantification of OCR confirmed a significantly higher ATP production, which could be related to the shift from the glycolysis to oxidative phosphorylation. This observed shift between the glycolytic and mitochondrial pathways, seen in the assay as opposite trends with respect to ECAR and OCR measurements, agrees with other reports^31,40^. The metabolic flux assay showed that the shift from proliferation to differentiation in keratinocytes is associated with a shift from glycolysis to oxidative phosphorylation. It should therefore be expected that proliferative and differentiating cells cultures, i.e., low and high Ca^2+^ respectively, will have different compositions of the fluorescent co-enzymes NADH and FAD, i.e. the target fluorophores for MPM-FLIM.

### MPM-FLIM detects changes consistent with a shift from glycolysis to oxidative phosphorylation in differentiating keratinocytes

**Fig. 3:**
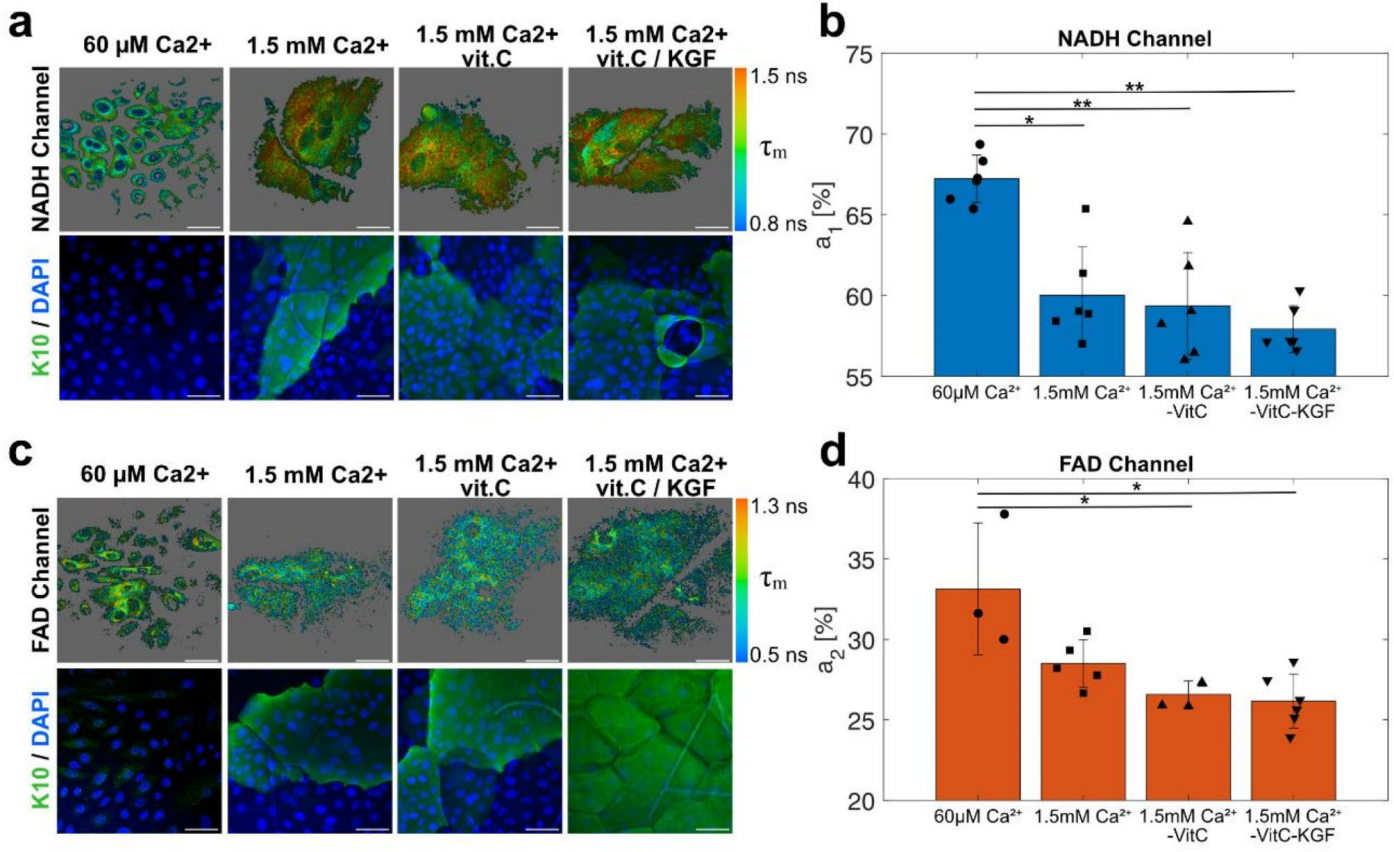
MPM-FLIM detects changes in cellular metabolic state after 96 h high calcium treatment. (**a**) *Upper panel*: FLIM images of live human keratinocytes in the NADH channel, colored by the pixelwise mean fluorescence lifetime (*τ*_*m*_). Background pixels excluded from FLIM analysis are colored grey. *Lower panel*: confocal immunofluorescence images of K10 and DAPI at the same imaging position taken after the MPM-FLIM imaging. Scale bar: 50 μm. (**b**) Mean and standard deviation of the fraction of the short fluorescence decay component (*a*_*1*_) in the NADH channel (*n* = 6). (**c**) *Upper panel*: FLIM images of live human keratinocytes in the FAD channel, colored by the pixelwise mean fluorescence lifetime (*τ*_*m*_). *Lower panel*: confocal immunofluorescence images of K10 and DAPI at the same imaging position taken after the MPM-FLIM imaging. Scale bar: 50 μm. (**d**) Mean and standard deviation of the fraction of the long fluorescence decay component (*a*_2_) in the FAD channel (3 ≤ *n* ≤ 6). For (b) and (d), markers indicate the value for the pooled fluorescence decays of each image. FLIM images were created using SPCImage (v9.88, https://www.becker-hickl.com/products/spcimage/) and post-processed with MATLAB (R2023b, https://www.mathworks.com/). Immunofluorescence images were created using ImageJ (v1.54, https://imagej.net/software/fiji/).

After validating our epidermal differentiation model with respect to keratin expression and metabolism, we then used the same model for metabolic imaging with MPM-FLIM. HEKn cells were imaged after 96 h treatments. The imaging was performed using the excitation wavelengths of 750 and 900 nm, targeting NADH and FAD respectively while minimizing the spectral crosstalk from keratin^24^. Images were aquired at the basal layer for cells grown at low Ca2 + and at the suprabasal layer for high Ca2 + treated cells. The samples were fixed immediately after imaging and stained for K10/DAPI. Immunofluorescence images were then acquired at approximately the same positions as for MPM. The mean fluorescence lifetime images (Fig. 3a and c, top panels) reveal a shift towards longer fluorescence lifetimes for the high Ca^2+^ treated cells in the NADH channel (750 nm excitation, 445/60 nm emission), as well as a shift to shorter lifetimes in the FAD channel (900 nm excitation, 580/150 nm emission). This finding is correlated with the presence of differentiated K10-positive cells in the immunofluorescence images for 1.5 mM Ca^2+^ cultures (Fig. 3a and c, bottom panels).

A biexponential decay model was required to adequately fit the pooled fluorescence decays of each image (Fig. S3). Fitting this model to the fluorescence decays in each field-of-view yielded a mean *τ*_1,NADH_ of 0.53 (± 0.02) ns and a mean *τ*_2,NADH_ of 2.53 (± 0.12) ns in the NADH channel and a mean *τ*_1,FAD_ of 0.41 (± 0.02) ns and a mean *τ*_2,FAD_ of 2.43 (± 0.06) ns in the FAD channel when averaged over all fields-of-view in this experiment. The fitted lifetimes of the fast and slow decay components were essentially insensitive to changes in treatment conditions (Fig. S4). Subsequently, observed changes in the mean fluorescence lifetime (*τ*_m_) were interpreted as changes in the relative fractions of the respectively decay components. The lifetimes of the fast decay component in the NADH channel (*τ*_1,NADH_) and the slow decay component in the FAD channel (*τ*_2,FAD_) are in line with expected values for free NADH (≈ 0.4 ns) and free FAD (≈ 2.5 ns) from the literature^41–49^ and our previous work^24^. The slow decay component in the NADH channel (*τ*_2,NADH_) and the fast decay component in the FAD channel (*τ*_1,FAD_) are within the lifetime range referred to in the literature as bound NADH and bound FAD respectively^41–49^.

To better compare the fractions of free NADH and FAD within each sample, the curve-fitting was repeated for each channel with *τ*_1_ and *τ*_2_ fixed to the mean values from the initial fit, such that only the pre-exponential factors *a*_1_ and *a*_2_ were allowed to vary. The relative percentages of *a*_1_ and *a*_2_ were compared for each treatment condition (Fig. 3b and d). The MPM-FLIM results show that the fractions of free NADH and FAD decrease in cells undergoing high calcium treatments. This is consistent with a shift from glycolysis to mitochondrial respiration for differentiated cells, as observed from the Seahorse assay. The shift from the glycolytic pathway to oxidative phosphorylation increases the oxidation of NADH to NAD^+^ in the mitochondrial complex I, reducing the NADH-related fluorescence. Additionally, the higher contribution of oxidative phosphorylation is expected to decrease the relative fluorescence contribution of free FAD. Therefore, the MPM-FLIM results indicate metabolic changes that are consistent with differentiation.

### Longitudinal study of keratinocyte differentiation with MPM-FLIM for 96 h

**Fig. 4:**
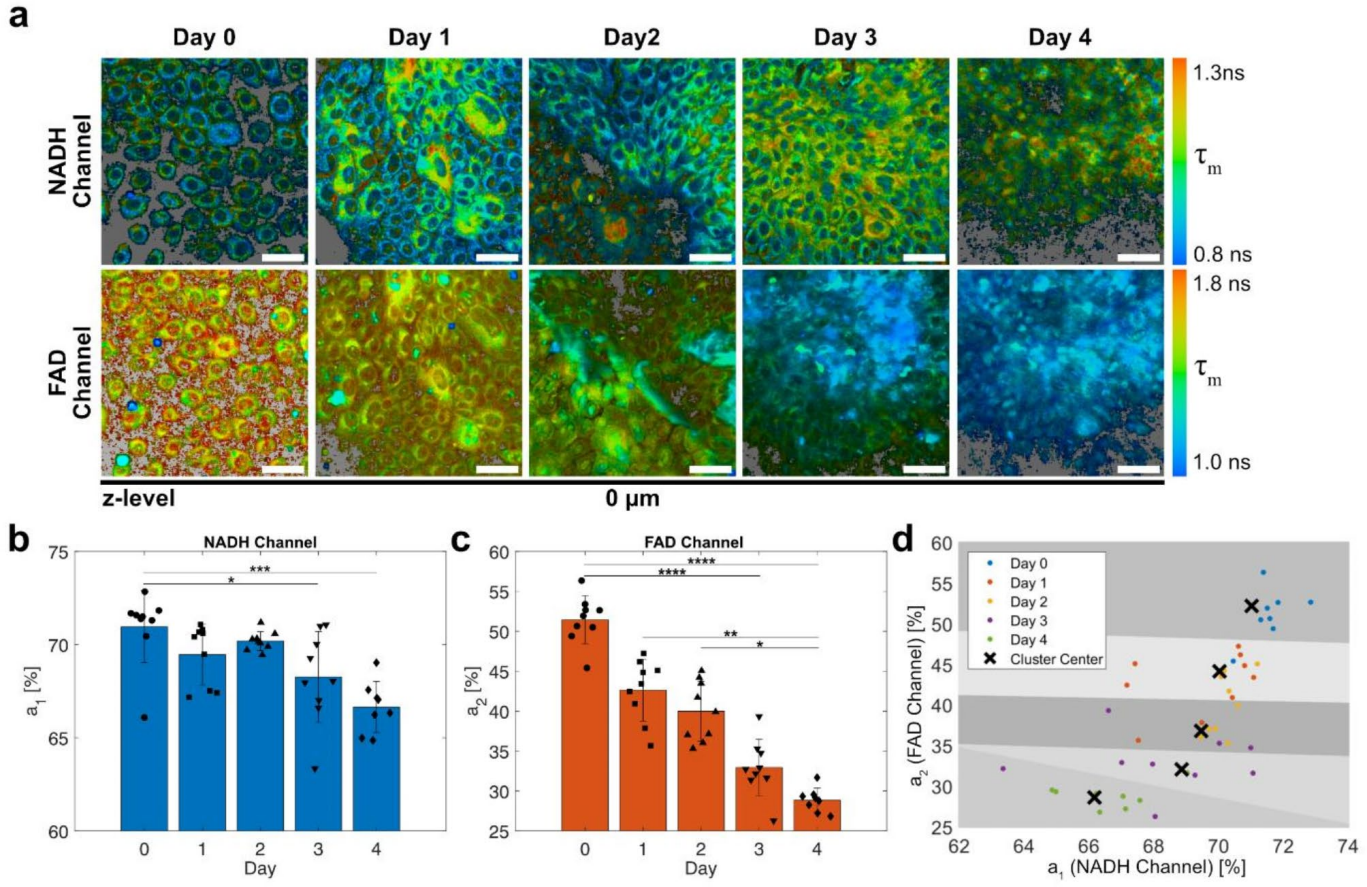
Following the shift in metabolic state in keratinocytes undergoing differentiation using MPM-FLIM. (**a**) FLIM-images of live human keratinocytes grown in high calcium over 4 days, colored by the pixelwise mean fluorescence lifetime (*τ*_*m*_). The basal layer of cells (z-level = 0 μm) are shown. Background pixels excluded from FLIM analysis are colored grey. Scale bar: 50 μm. (**b**,** c**) Mean and standard deviation of the relative fraction of (**b**) the short fluorescence decay component (*a*_*1*_) in the NADH channel and (**c**) the slow fluorescence decay component (*a*_*2*_) in the FAD channel. Markers indicate the value for the pooled fluorescence decays of each image. 8 ≤ *n* ≤ 9 over 3 separate culture dishes. (**d**) Scatter plot of *a*_*1*_ (NADH channel) and *a*_*2*_ (FAD channel) at each imaging position. The shaded background represents the clusters as computed by k-means clustering (*k* = 5; distance measure: Euclidean), with crosses marking the cluster centers. Images were created using SPCImage (v9.88, https://www.becker-hickl.com/products/spcimage/) and post-processed with MATLAB (R2023b, https://www.mathworks.com/).

Following our confirmation that high calcium treatment induces HEKn cell differentiation and that MPM-FLIM can detect the associated metabolic changes (Fig. 3), we proceeded to monitor this process over a 96 h period using an on-stage incubation system. As before, HEKn cells were cultured in regular growth medium (60 µM Ca^2+^) to reach higher confluence. Thereafter, the growth medium was replaced with a high calcium medium (1.5 mM Ca^2+^). The cells were imaged in the same position at 750 nm and 900 nm excitation immediately after the medium change (Day 0) and every day for the following 4 days (Fig. 4a). The samples were exchanged to fresh high calcium media at the end of imaging on Day 2. On day 4, the samples were imaged at 3 z-levels, which showed that the suprabasal layer had formed (Fig. S5a). Over time, the cells located basally created a homogenous densely packed layer, while the suprabasal cells were larger in size and exhibited higher fluorescence intensity. We performed FLIM analysis using the same approach as in the previous experiment. The mean *a*_1_ and *a*_2_ followed the expected trend for a shift towards oxidative phosphorylation and indicated a decrease in the fluorescence fraction of free NADH and free FAD over the 4-day period (Fig. 4b and c). The suprabasal layers imaged on day 4 showed a decrease in *a*_1,NADH_ and an increase in *a*_2,FAD_ compared to the basal layer (Fig. S5b and c), although this difference was found not statistically significant.

Based on the data presented in Fig. 4a–c, we reasoned that the information obtained from MPM-FLIM could be used for the classification of HEKn differentiation status. As a proof of concept, we performed a discrimination analysis based on k-means clustering (*k* = 5) using the Euclidean distance between the samples as represented by *a*_1,NADH_ and *a*_2,FAD_ (Fig. 4d). Notably, the mode of each cluster corresponded to each day of the experiment. Two of the clusters contained only data acquired from a single day (day 0 and day 3), while the other clusters contained only data also from adjacent days (*mode* ± 1). Overall, the k-means clusters corresponded well to the trend observed in the experimental data. Metabolic imaging using MPM-FLIM of HEKn cells therefore provides useful information that could potentially be used for an automated classification scheme for characterizing status of keratinocyte differentiation based on classical machine learning or deep learning models.

## Discussion

The results from this study demonstrate that MPM-FLIM using two different excitation wavelengths, i.e. 750 nm targeting NADH and 900 nm targeting FAD, can be applied as tool to monitor keratinocyte differentiation using FLIM as a readout of changing metabolites. Using a biexponential fitting model, the FLIM-data show a decreased relative fraction of intracellular NADH and FAD with increasing Ca^2+^ levels, corresponding to different stages of differentiation and increased oxidative phosphorylation as confirmed by the biochemical assays. The keratinocytes cultured at 1.5 mM Ca^2+^ exhibited increased expression of differentiation markers (K1/K10) and production of K1 and K10 proteins, as compared to 60 µM Ca^2+^, in agreement with literature^35,50^. Furthermore, a metabolic shift from glycolysis to oxidative phosphorylation was confirmed to be associated with differentiation for the cells at high calcium treatment. This observation provides evidence that epidermal differentiation follows a similar metabolic trend to that established in other cell types, in which proliferating cells prefer glycolysis but differentiated cells shift towards oxidative phosphorylation. The metabolic shift can be understood in the context of the Warburg effect, which was originally observed in cancer cells as the ability to promote glycolysis even in the presence of oxygen^51^, but is now also shown to also play a role in proliferating cells^52,53^, despite the fact that glycolysis is a less efficient pathway for ATP generation compared to oxidative phosphorylation^54^. So far, there is no definitive explanation for the preference for glycolysis in proliferating cells, but it is speculated to be driven by the need to generate biomaterials to fuel cell expansion and division^55^. Regardless, the metabolic shift we observed conforms to the trend reported in the literature^27–31^ and provides the rationale for exploring MPM-FLIM as a non-destructive tool in this context.

In this study, a 2D tissue culture model was chosen for its robustness and simplicity. Even if a fully developed 3D model (e.g. one using an air interface technique^56^) would have better resembled the true layered structure of the skin, a fully developed layer structure would have complicated the comparison between MPM-FLIM and the biochemical analysis. Although the aim was to culture the cells in a monolayer, the differentiated cell pool at 1.5 mM Ca^2+^ was found to be heterogenous, where the K10-positive cells were located above the basal layer of K14-positive cells, (e.g., see Fig. 1 as enlarged cells marked for K10 in green). At this stage, the differentiation stage likely represents the transition between *stratum basale* and *stratum spinosum*. While slight enlargement of the cells was visible in the images (see Fig. 1: K10-positive cells in green in 1.5 mM Ca2 + treatment), the terminal stage of differentiation and formation of *stratum granulosum* and *stratum corneum* did not take place. In the subsequent MPM-FLIM experiments, high calcium treatment generated a decrease in the fluorescence lifetime fractions *a*_1,NADH_ and *a*_2,FAD_ corresponding to NADH and FAD respectively, consistent with increased mitochondrial respiration in differentiating HEKn cells. We note that that the deviations in absolute values of *a*_1,NADH_ and *a*_2,FAD_ between Figs. 3 and 4, are possibly related to the differences in cell density. Thus the focus of the analysis should be comparing the trends in each figure. Through *k*-means clustering, we showed that it was possible to classify the MPM-FLIM data into clusters that essentially correspond to each day of the experiment. Overall, the results confirm the feasibility of MPM-FLIM as an approach for monitoring the tissue culture for several days (up to 96 h).

It should be noted that the suprabasal layers in the confocal immunofluorescence images (Fig. S2) have a different appearance compared to those in the MPM-FLIM images (Fig. S5). This is likely because the confocal images are staining and imaging nuclei (DAPI), K10 and Dsg3, while the MPM-FLIM signal originates primarily from NADH and FAD. Thus the intracellular distribution of the fluorescence signals are expected to be different. The MPM-FLIM images also contain more cell debris due to it being a live culture whereas the confocal images were acquired after fixation and immunostaining.

At this stage, we chose to analyze the FLIM data with respect to whole images, as the biochemical data validating the cell model was acquired for cell ensembles. However, as seen in Fig. 4a, the cells also show some heterogeneity in fluorescence lifetime, such as on Day 1 and 2 in the NADH channel and day 3 in the FAD channel. Cell specific analysis and automatized image segmentation is a future direction that can be implemented to further strengthen the analysis and improve the sample statistics. Our experience is that while current state-of-the-art cell segmentation algorithms such as Cellpose^57,58^ seem to work reasonably well for segmenting keratinocytes in the proliferating/early differentiated stages, they fail to segment keratinocytes at later stages of differentiation, for which the cytoplasmic boundaries are not as clear in the MPM-FLIM data. When it comes to segmenting nuclei, current models trained to segment labelled nuclei^57–59^ are insufficient for the more challenging label-free MPM images. Development of better models for segmenting MPM images of keratinocytes would facilitate analysis at the cell level.

As a method for visualization of live cells and tissues, MPM-FLIM has the advantage of being label-free and non-invasive with limited phototoxicity. However, tissue autofluorescence is complex and influence from other factors, such as fluorescence from structural proteins and oxidation products^24,60^, should be considered. The excitation wavelengths of 750 nm and 900 nm were chosen here to minimize spectral crosstalk from keratin. Even so, contribution of keratin and/or other intrinsic fluorophores cannot be excluded. Interestingly, the spectral cross-talk from additional components also presents an opportunity to acquire and extract additional information from a single experiment by elaborating on the data-analysis. It should be acknowledged that regression fitting of complex fluorescence decays with 3 or more exponential components remains demanding on photon count and signal-to-noise^11^, which can be challenging when acquiring autofluorescence from living cells. It is possible that fit-free methods, such as phasor-FLIM^12,13^ or those based on deep-learning^61^ could complement or surpass fit-based methods in low photon count situations, but their effectiveness in this aspect remains to be fully tested. Potentially, the inclusion of other fluorescence and imaging parameters such as intensity, cell morphology and texture analysis would likely improve the accuracy of a classifier algorithm. With the recent rapid advance of deep-learning models in biological image segmentation and analysis, one could envision a fully automated classifier that could be trained to identify epidermal differentiation status from MPM-FLIM data. Another possible challenge is the acquisition speed. For the type of investigative study as presented in this paper, the imaging speed of around 1 to 2 min per image used here does not pose any major issues; however, if the approach is to be implemented for large scale tissue culturing the data acquisition time is likely to hamper the applicability of the method. Apart from developing methods that require a lower photon count to accurately estimate the lifetime, image upsampling has been proposed as a possible approach to improve acquisition speed^62^.

Taken together, the results presented here provide evidence that MPM-FLIM data can be linked to the metabolic status of keratinocytes at different stages of differentiation. Using discrimination analysis, we show that fluorescence lifetimes parameters correlated with tissue culture status over several days, offering a non-destructive tool for quality assurance. Future work should include implementing image analysis tools to enable cell-specific analysis, alongside machine learning models that can classify the metabolic or differentiation status of individual cells. Incorporating additional fluorescence and imaging parameters, such as intensity and cell morphology, combined with and a deeper understanding of the contribution from other tissue constituents such as keratin, will likely improve the accuracy of this technique. Ultimately, these improvements could lead to an automated scheme for non-invasive monitoring of complex organotypic cell culturing.

## Materials and methods

### Cell culture

Neonatal human epidermal keratinocytes (HEKn) were purchased from Thermo Fisher Scientific.

HEKn cells were cultured in Keratinocyte Growth Medium 2 (KGM2, Promocell) supplemented with 60 µM of Ca^2+^, 0.2% gentamicin/amtmphotericin (G/A, Gibco). The addition of the supplement mix to KGM2 provides 4 µl/ml bovine pituitary extract, 0.125 ng/ml epidermal growth factor (recombinant human), 5 µg/ml insulin (recombinant human), 0.33 µg/ml hydrocortisone, 0.39 µg/ml epinephrine, and 10 µg/ml transferrin (recombinant human). The cells were seeded at the density 2.5 × 10^3^ cells/cm^2^ in a T-25 flask in 5 ml of supplemented growth medium and incubated in a humidified cell culture incubator at 37 °C and 5% CO_2_. The growth medium was changed every other day until 50% confluence and every day thereafter. The cells were subcultured at 80% confluence.

Prior to analysis, HEKn cells were seeded at the cell density 5 × 10^3^ cells/cm^2^ and grown for 96 h in the following conditions: (1) regular KGM2 (60 µM Ca^2+^), (2) KGM2 supplemented with 1.5 mM Ca^2+^, (3) KGM2 supplemented with 1.5 mM Ca^2+^ and 50 µg/ml vit. C, and (4) KGM2 supplemented with 1.5 mM Ca^2+^, 50 µg/ml vit. C and 10 ng/ml keratinocyte growth factor (KGF). Growth medium was replenished after 48 h. The cells were then collected for analysis.

### qPCR

The cells were grown in 6-well plates. For each RNA sample, cell material from two wells was combined into one sample to yield a higher RNA concentration. RNA extraction was carried out using the RNeasy Plus Mini Kit (QIAGEN) according to the protocol supplied by the manufacturer. RNA concentration of each sample was measured with a NanoDrop 2000 (Thermo Fisher Scientific). cDNA was synthesized from 1 µg of RNA using the RevertAid H Minus First Strand cDNA Synthesis Kit (ThermoFisher Scientific). qPCR was performed in a BioRad CFX Connect Real Time system and using PowerUp SYBR Green Master Mix (Applied Biosystems). The relative gene expression of keratins was calculated by the ddCT method, using YWHAZ as the reference gene^63^.

Pre-designed KiCqStart primers (Merck) were purchased for the study:

- YWHAZ:
  - 5ʹ-AACTTGACATTGTGGACATC-3ʹ.
  - 5ʹ-AAAACTATTTGTGGGACAGC-3ʹ.
- K5:
  - 5ʹ-AGTTTGTGATGCTGAAGAAG-3ʹ.
  - 5ʹ-GTTAATCTCATCCATCAGTGC-3ʹ.
- K14:
  - 5ʹ-AGATCAAAGACTACAGTCCC-3ʹ.
  - 5ʹ-ACTCTGTCTCATACTTGGTG-3ʹ.
- K1:
  - 5ʹ-GAGGATATAGCCCAGAAGAG-3ʹ.
  - 5ʹ-ATCTAAGTCTCTGGATCACAC-3ʹ.
- K10:
  - 5ʹ-GGAGATAGAACTACAGTCCC-3ʹ.
  - 5ʹ-GTATTCAGTATTCTGGCACTC-3ʹ.
- K6A:
  - 5ʹ-TGAGGATGAAATCAACAAGC-3ʹ.
  - 5ʹ-TTCAACCTTGTTCATGTAGG-3ʹ.

### Western blot

The cells were grown in 6-well plates. The cells were placed on ice, washed three times with ice-cold PBS, and lysed with 100 µl of ice-cold lysis buffer (50 mM HEPES at pH 7.2, 90 mM KCl, 0.5% Igepal) containing Protease Inhibitor Cocktail (P8340, Sigma). The lysate was scraped down, moved to Eppendorf tubes, and centrifuged at 7000 rpm at 4 °C for 5 min in a microcentrifuge. Pierce BCA Protein Assay kit (ThermoFisher Scientific) was used for determination of protein concentration in each sample, where 10 µl of the lysate was used for an assay performed in 96-well plate. The post nuclear supernatant was then stored at – 20 °C until analysis. Samples were diluted in SDS sample buffer, heated for 2 min. at 96 °C and resolved on a 10% acrylamide gel.

For standardization of protein levels, gels were loaded with equal protein levels (acc. to the Pierce BCA Assay kit readout), proteins separated according to the size with electrophoresis, the gels were then stained with Coomassie blue, and lane intensities were quantified using ImageJ^64,65^. Following loading adjustments according to Coomassie blue stain to equalize protein levels, resolved proteins were transferred to nitrocellulose membranes overnight using a wet transfer system.

Nitrocellulose membranes were briefly stained with Ponceau stain, washed in 0.1% PBS-Tween, and blocked in 5% milk in PBS for 30 min. Membranes were then incubated for 1 h with primary antibodies diluted in blocking milk, washed three times in 0.1% PBS-Tween for 5 min, followed by a 1 h incubation with secondary antibodies diluted in blocking milk. The membranes were imaged using chemiluminescence.

Primary antibodies used were Anti-Cytokeratin 5 (ab52635, Abcam), Anti-Cytokeratin 14 (ab7800, Abcam), Anti-Cytokeratin 1 (ab185628, Abcam), Anti-Cytokeratin 10 (ab76318, Abcam), Anti-Cytokeratin 6 (ab93279, Abcam) and Anti-β-actin (AC15, Sigma). Secondary antibodies were Anti-Mouse IgG (whole molecule, A4416, Sigma) and Anti-Rabbit IgG (whole molecule, A6154, Sigma).

### Immunofluorescence

Cells were grown on sterilized glass coverslips (10 mm, #1.5) placed in 6-well plates, washed three times in PBS complete (37 °C), fixed in 4% formaldehyde in PBS for 10 min and then permeabilized in 0.2% Triton X100 in PBS for 1 h. The cells were then incubated for 30 min. in 3% bovine serum albumin (BSA) diluted in PBS and incubated for 1 h in a primary antibody diluted in 3% BSA, followed by three washes in PBS, and 1 h incubation with a corresponding secondary antibody diluted in 3% BSA. The cells were then washed three times in PBS and incubated for 10 min. in 10 µg/ml DAPI (11530306, Invitrogen). The coverslips were washed three times in PBS, once in MilliQ water, and mounted with 5 µl of Prolong Gold on a microscope slide. The samples were allowed to set overnight at RT and were stored at 4 °C in the dark thereafter.

Primary antibodies were Anti-Cytokeratin 5 (ab52635, Abcam), Anti-Cytokeratin 14 (ab7800, Abcam), Anti-Cytokeratin 10 (ab76318, Abcam) and Anti-Desmoglein-3 (ab231309, Abcam). Secondary antibodies were Goat Anti-Rabbit IgG H&L Alexa Fluor 488 (ab150077, Abcam) and Goat Anti-Mouse IgG H&L AlexaFluor 597 (ab150116, Abcam).

### Metabolic flux assay

Real-time measurements of oxygen consumption rate (OCR) and extracellular acidification rate (ECAR) were performed on the Seahorse XFe96 Extracellular Flux Analyzer (Agilent Technologies), using XF Cell Mito Stress Test and XF Glycolysis Stress Test kits (Seahorse Biosciences), respectively. Prior to the experiments, cell seeding density, as well as the optimal concentrations of carbonyl cyanide-4-(trifluoromethoxy) phenylhydrazone (FCCP) and oligomycin, were determined empirically. The cells were seeded in 96-well plates (XF96 cell culture microplates, Agilent) at a cell density 1 × 10^4^ cells per well and grown for 96 h.

The day prior to the experiment, 200 µl of XF calibration media was added to the XF sensor cartridges and kept in a non-CO_2_ incubator overnight. For ECAR assessment using XF Glycolysis Stress Test, on the day of the experiment the culture medium was replaced with 180 µl of XF DMEM supplemented with 1 mmol/l sodium pyruvate, and 2 mmol/l glutamine and incubated for 1 h. The ports of the XF sensor cartridges were loaded with the test compounds (glucose, 10 mM; oligomycin, 1 µM; 2-deoxyglucose, 50 mM), system was calibrated and ECAR in the cells was measured.

For the OCR assessment with a XF Cell Mito Stress Test, the culture medium was replaced with 180 µl of XF DMEM supplemented with 10 mmol/l glucose, 1 mmol/l sodium pyruvate, and 2 mmol/l glutamine and incubated for 1 h. The ports of the XF sensor cartridges were loaded with the test compounds (oligomycin, 1 µM; FCCP, 1 µM; Rotenone/AA, 0.5 µM), the system was calibrated and OCR in the cells was measured.

All data were normalized to the spectrophotometric readout using Pierce BCA assay, directly proportional to the protein concentration.

### Confocal laser scanning microscopy

A commercial LSM 710 NLO (Carl Zeiss, Jena, Germany) was used for confocal imaging and spectral MPM. A water-immersion objective Plan-Apochromat 20× (NA 1.0) was used for live cell imaging.

An Ar-laser (488 nm) was utilized for Alexa 488 conjugated secondary antibodies (497–597 nm emission), a laser diode (561 nm) for Alexa 594 nm (577–758 nm emission), and a laser diode 405 nm for DAPI (497–577 nm emission).

### Multiphoton fluorescence lifetime imaging

MPM-FLIM was performed using an experimental MPM inverted system, as described in the previous work^24^. Cells were imaged using glass bottom dishes with a grid (ibidi, #1.5, Ø = 35 mm) to facilitate localization on different instruments. The cultures were maintained at 37 °C and 5% CO_2_ using a stage top incubator (ibidi). A fs-pulsed NIR laser (MaiTai DeepSee, Newport Spectra-Physics) was set to operate at two excitation wavelengths: 750 nm and 900 nm. The laser power was kept at approximately 20 mW, as measured at the sample, controlled by an external acousto-optic modulator (MT110-B50A1.5-IR-Hk, AA Opto-Electronic). A water immersion objective (40×, 0.8 C “Achroplan” NIR, Carl Zeiss) was used. The detection was performed by two separate GaAsP detectors (H7422P-40 MOD, Hamamatsu), interfaced to two time-correlated single-photon counting (TCSPC) modules (SPC 150, Becker & Hickl), enabling FLIM acquisition. Two spectral combinations were enabled by utilizing the dichroic mirror (509 nm cut off, Semrock Inc) combined with filter combinations in 445/60 nm and 580/150 nm (Semrock, Brightline), for detection of NADH and FAD emission respectively. The recording of the TCSPC fluorescence data was done using the SPCM64 software (Becker & Hickl). The imaging acquisition time was approximately 60 s and 80 s for each image plane at 750 and 900 nm, respectively.

### Image processing and analysis

For confocal and spectral microscopy images, image processing and spectral analysis were done using ImageJ^64,65^ (U.S. National Institutes of Health, Bethesda, Maryland) and MATLAB (vR2020b, MathWorks Inc).

MPM-FLIM images were analyzed by SPCImage software (v9.88, Becker & Hickl). The instrument response function (IRF) was estimated using the ‘Auto IRF’ setting in the software and had a full-width half maximum of approximately 300 ps. The settings were validated by fitting the fluorescence decay of Rhodamine B solution to obtain a lifetime of 1.3 ns, in good agreement with the literature^66^. For the whole-image analysis, an intensity threshold specific to each image to exclude dark background regions from the FLIM analysis. Additionally, for each image, the summed photon decay histograms were used for regression fitting.

A biexponential decay model was chosen for modeling fluorescence decay obtained from the cells,

1 $$\:f\left(t\right)={a}_{0}+{{a}_{1}\:e}^{-\text{t}/{\tau\:}_{1}}+{{a}_{2}\:e}^{-\text{t}/{\tau\:}_{2}}$$

where *τ*_1_ and *τ*_2_ correspond to the lifetimes of the fast and slow lifetime components respectively. The pre-exponential factors $\:{a}_{1}\:$and $\:{a}_{2}\:$describe the amplitude components of the fast and slow decay, respectively. A minimum intensity threshold was applied to each image to select a region of interest (ROI) containing the cellular autofluorescence signal. An initial fit to the biexponential decay model was carried out using the pooled fluorescence decays of all pixels within the ROI. To increase comparability of the fit results within an experiment, the curve-fitting was repeated while fixing *τ*_1_ and *τ*_2_ to the respective their mean values from all fields-of-view of an experiment in the initial fit. This fit was applied to both the pooled fluorescence decays within each ROI and to the pixelwise decays. The pre-exponential factors were allowed to fit freely and used for comparison and further analysis. Figure plotting and k-means clustering were performed using MATLAB (v2023b, MathWorks Inc).

### Statistical analysis

Statistical analysis of the data was performed using GraphPad Prism (GraphPad Software, San Diego, CA, USA). Statistical testing of qPCR results was done using Brown-Forsythe and Welch ANOVA followed by Dunnett’s T3 multiple comparisons test. Statistical testing for the XF Searhorse assays and the fit results of MPM-FLIM was done using the Kruskal-Wallis test followed by Dunn’s multiple comparisons test. Results were considered statistically significant when *p* < 0.05.

## Electronic supplementary material

Below is the link to the electronic supplementary material.

Supplementary Material 1

## Data Availability

All data and analysis scripts are available on Zenodo: 10.5281/zenodo.11189910.
