# Supplementary material for: Insights into metabolic changes during epidermal differentiation as revealed by multiphoton microscopy with fluorescence lifetime imaging
Source: Sci Rep. 2025 Feb 21;15:6377. doi: 10.1038/s41598-025-90101-4 (PMC11845624; doi:10.1038/s41598-025-90101-4)
Supplement: Supplementary file 1 — Supplementary Material 1 [file 41598_2025_90101_MOESM1_ESM.pdf]

Supplementary Information

Insights into metabolic changes during epidermal differentiation as revealed by multiphoton microscopy with fluorescence lifetime imaging

Monika Malak, Chen Qian*, Jeemol James, Syam Nair, Julie Grantham, and Marica B. Ericson*

* Corresponding authors: e-mail: chen.qian@gu.se, marica.ericson@gu.se.

Supplementary Figure S1: Effect of high calcium treatment and supplementation with vitamin C and/or KGF on the expression of keratin isoforms.

Supplementary Figure S2: Differentiated cells are located suprabasally.

Supplementary Figure S3: A biexponential decay model describes the fluorescence decay of keratinocytes.

Supplementary Figure S4: Change in fluorescence decays of keratinocytes after 96h high calcium treatment.

Supplementary Figure S5: MPM-FLIM of suprabasal layers on after 96h high calcium treatment.

Supplementary Figure S6: Full western blots of keratinocyte cultures.

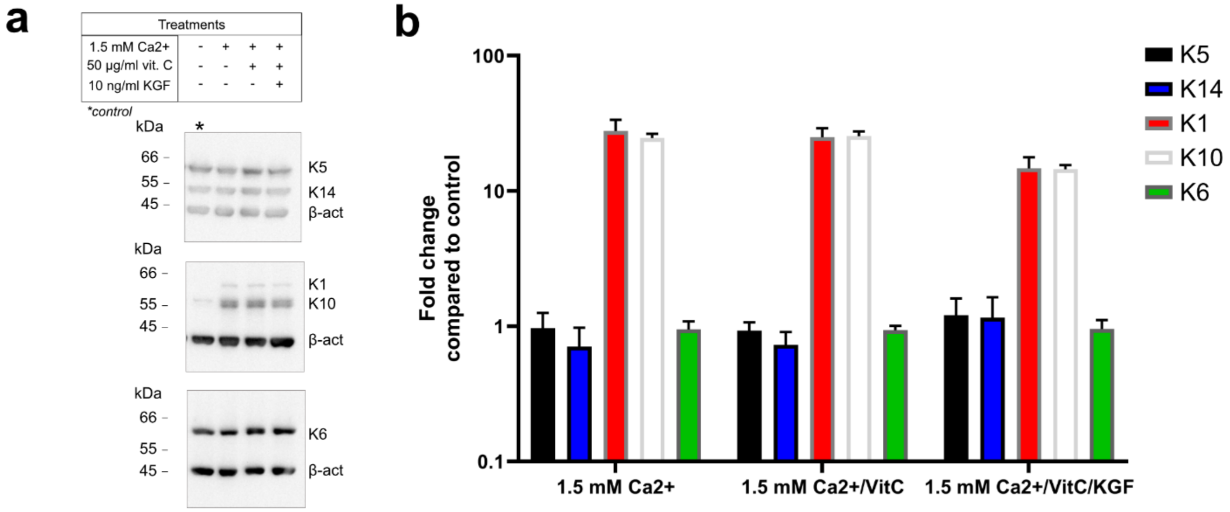

c

Comparison		K5	K14	K1	K10	K6
60 µM Ca^{2+}	1.5 mM Ca^{2+}	ns	ns	****	****	ns
60 µM Ca^{2+}	1.5 mM Ca^{2+}/vitC	ns	ns	****	****	ns
60 µM Ca^{2+}	1.5 mM Ca^{2+}/vitC/KGF	ns	ns	****	****	ns
1.5 mM Ca^{2+}	1.5 mM Ca^{2+}/vitC	ns	ns	ns	ns	ns
1.5 mM Ca^{2+}	1.5 mM Ca^{2+}/vitC/KGF	ns	ns	****	****	ns
1.5 mM Ca^{2+}/vitC	1.5 mM Ca^{2+}/vitC/KGF	ns	ns	***	****	ns

Fig. S1. Effect of high calcium treatment and supplementation with vitamin C and/or KGF on the expression of keratin isoforms. (**a**) Changes in levels of K5, K14, K1, K10 and K6 in keratinocytes grown in (*columns from left to right*): control grown in 60 µM Ca^{2+} growth medium, 1.5 mM Ca^{2+} growth medium, 1.5 mM Ca^{2+} growth medium supplemented with vitamin C, and 1.5 mM Ca^{2+} growth medium supplemented with vitamin C and KGF, analyzed by western blotting. Primary antibodies were incubated simultaneously, as were the secondary antibodies. Protein loadings were standardized for total protein content based on Pierce™ BCA Protein Assay, Coomassie blue stained gels and densitometric analysis. An antibody to β actin was used on the same membrane to demonstrate even loading. Images of the full blots are presented in Fig. S6. (**b**) qPCR measurement of the expression of K5, K14, K1, K10 and K6 in keratinocytes treated with (*left to right*): 1.5 mM Ca^{2+}, 1.5 mM Ca^{2+} supplemented with vitamin C, 1.5 mM Ca^{2+} supplemented with vitamin C and KGF. Values are expressed as fold change compared to control cells grown in 60 µM Ca^{2+}. YWHAZ was used as a reference gene. Bar graphs represent mean ± SEM from 3 independent experiments. (**c**) Statistical comparison of qPCR results using Brown-Forsythe and Welch ANOVA with Dunnett's T3 multiple comparisons test. * $p \leq 0.05$, ** $p \leq 0.01$, *** $p \leq 0.001$, **** $p \leq 0.0001$. n = 12 over 3 independent repeats.

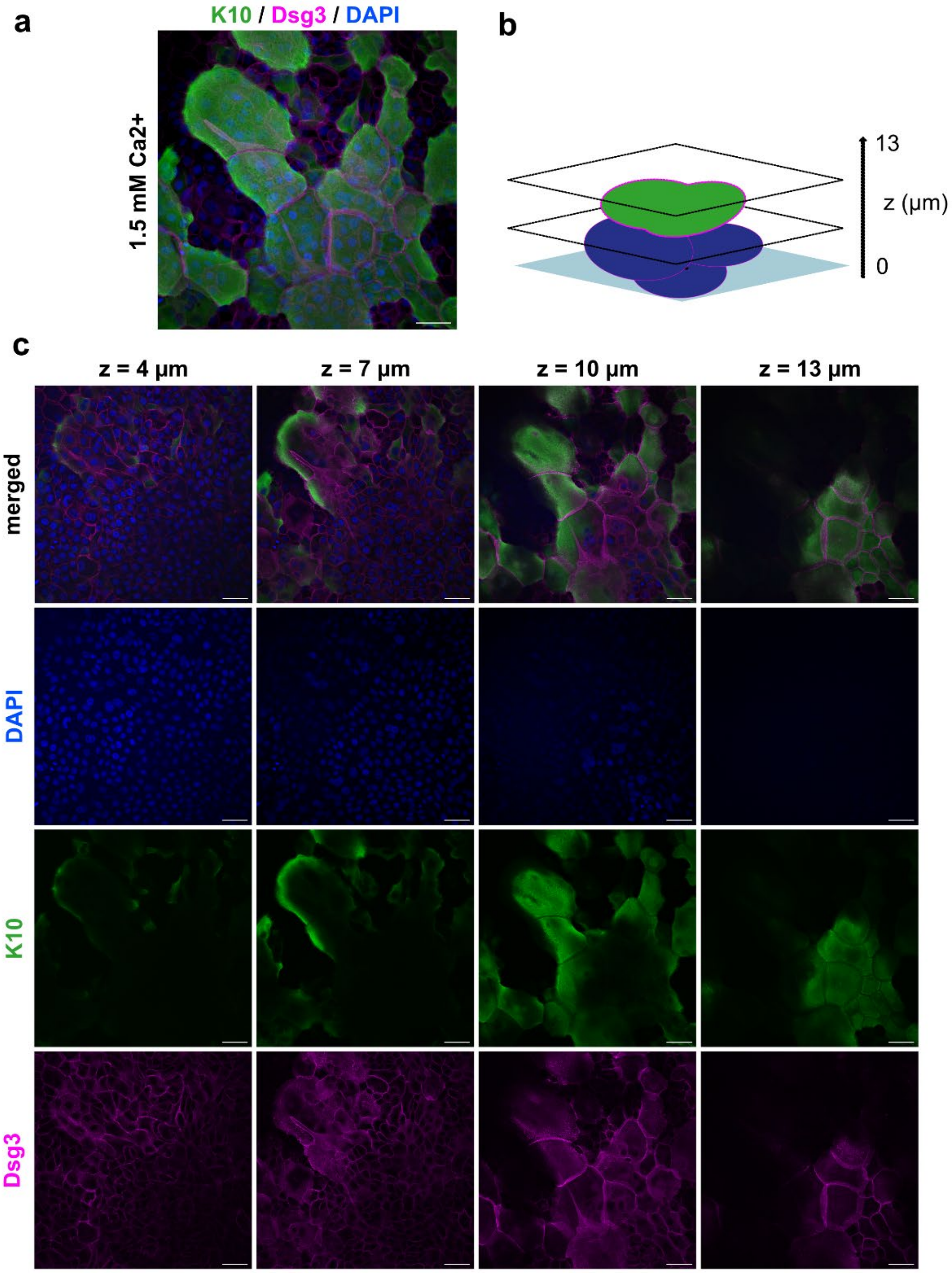

Fig. S2. **Differentiated cells are located suprabasally.** (**a**) Z-projection of confocal microscopy images of keratinocytes treated with 1.5 mM Ca^{2+}, taken at a step size of 0.65 µm. (**b**) Corresponding schematic drawing presenting the dimensionality of the sample and the image collection. (**c**) Images collected at four different z-levels (assuming the cover slip the cells were grown on to be at z = 0 µm, left to right): z = 4 µm, z = 7 µm, z = 10 µm, and z = 13 µm. The following panels are presented horizontally: merged image of all channels from each z-level, DAPI stain, K10 stain and Dsg3 stain. Scale bar: 50 µm. Images were created using ImageJ (v1.54, https://imagej.net/software/fiji/).

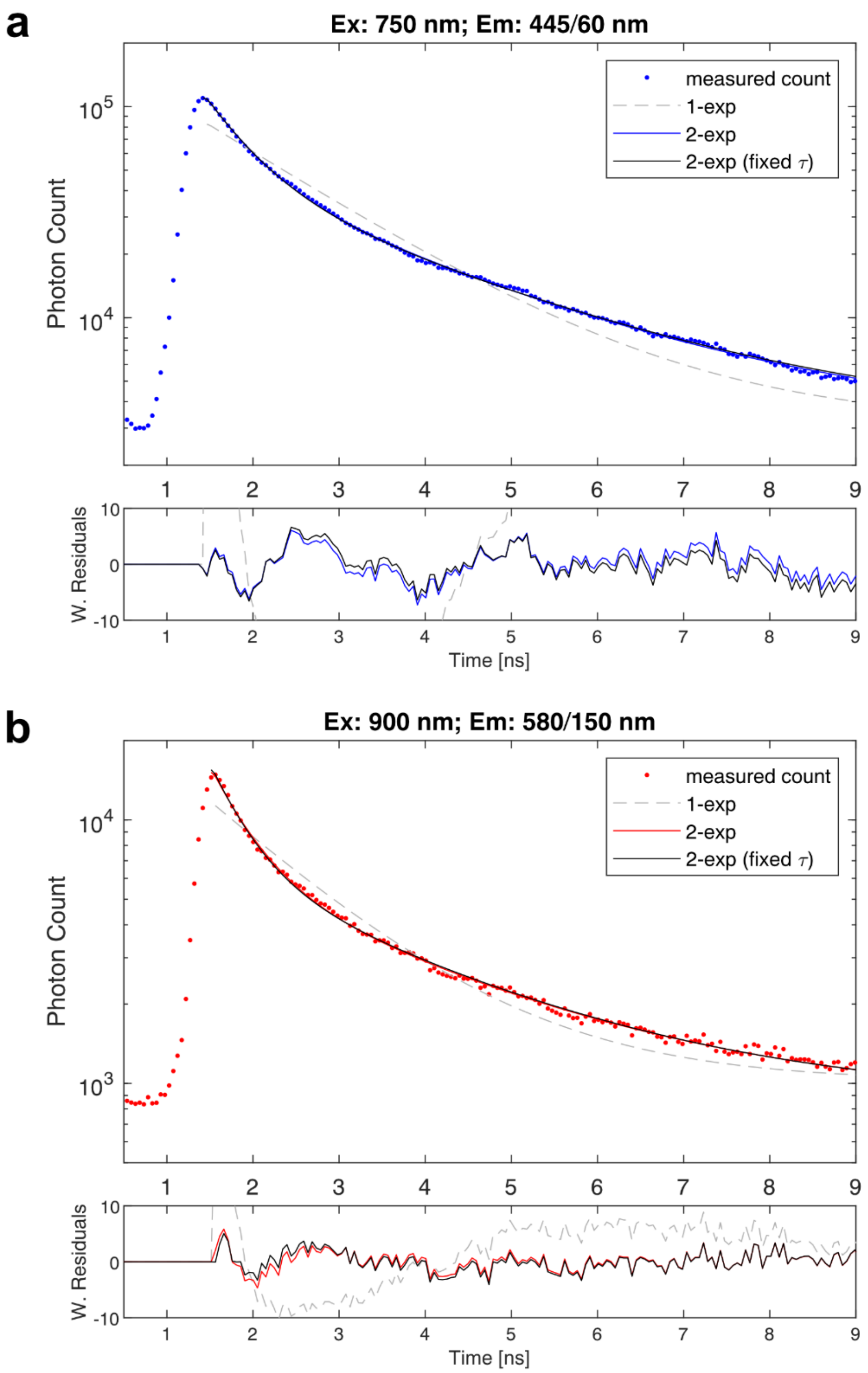

Fig. S3. A biexponential decay model describes the fluorescence decay of keratinocytes. (**a**) and (**b**) Examples of the pooled fluorescence decay of an image (*blue or red dots*) and the weighted-least-squares fit of a monoexponential decay model (grey dashed line), a biexponential decay model (*blue or red line*), and biexponential decay model with τ1 and τ2 fixed to the mean values of the dataset (*black line*). The corresponding weighted residuals are shown below each plot.

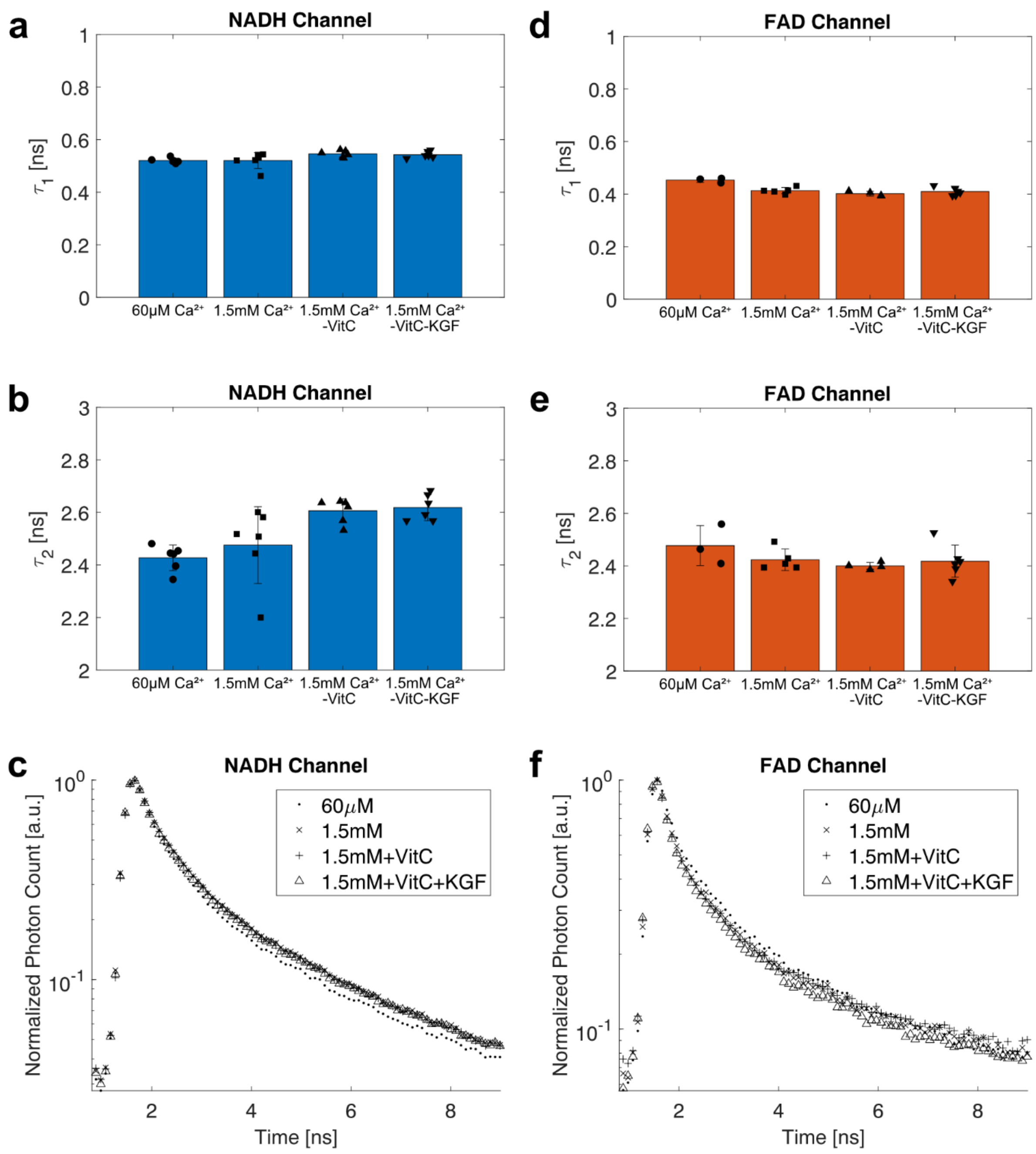

Fig. S4. Change in fluorescence decays of keratinocytes after 96h high calcium treatment. (a-b) Mean and standard deviation of the lifetimes of the (a) fast and (b) slow decay components in the NADH channel. Markers indicate the value for the pooled fluorescence decay of each image. (**c**) Pooled fluorescence decays of images in Fig. 3a. (**d-e**) Mean and standard deviation of the lifetimes of the (d) fast and (e) slow decay components in the FAD channel. (**f**) Pooled fluorescence decays of images in Fig. 3b.

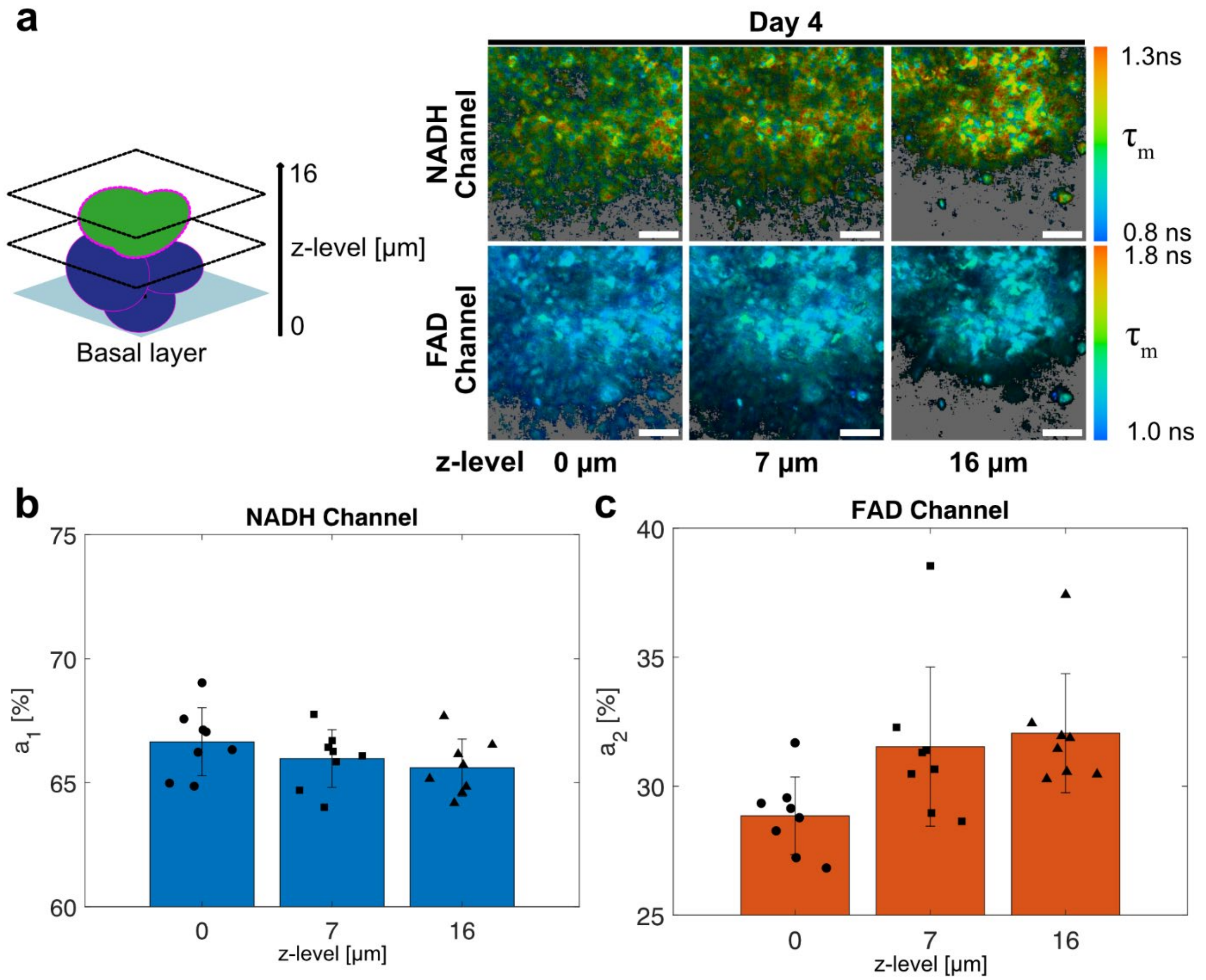

Fig. S5. MPM-FLIM of suprabasal layers after 96h high calcium treatment. (**a**) FLIM images of human keratinocytes at different scanning depths (z-levels) as shown in the schematic. False-color images of the pixelwise mean fluorescence lifetime. Background pixels excluded from FLIM analysis are colored grey. Scale bar: 50 µm. (**b-c**) Mean and standard deviation of the relative fraction of (b) the short fluorescence decay component (a_1) in the NADH channel and (c) the slow fluorescence decay component (a_2) in the FAD channel. Markers indicate the value for the pooled fluorescence decays of each image. Images were created using SPCImage (v9.88, https://www.becker-hickl.com/products/spcimage/) and post-processed with MATLAB (R2023b, https://www.mathworks.com/).

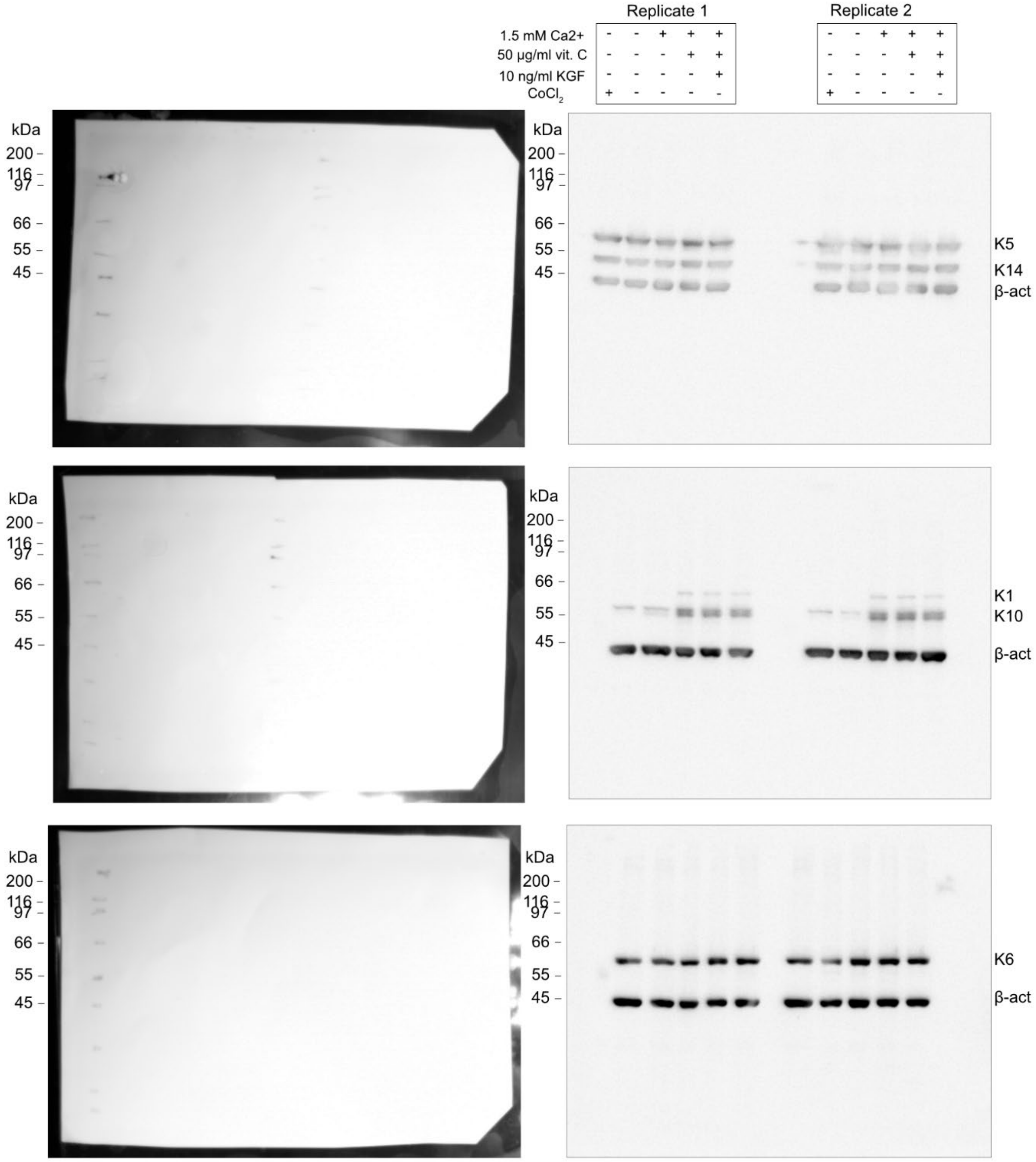

Fig. S6. Full western blots of keratinocyte cultures. *Left panels*: photographs of nitrocellulose membranes following transfer. Ladder positions were marked in pencil after Ponceau staining. *Right panels:* the corresponding full chemiluminescence images of the membranes after incubation with the respective primary and secondary antibodies. The treatment with $CoCl_2$, present on the membrane, should be disregarded as it was later deemed irrelevant for the work presented in this manuscript.
